# Performance optimisation of a holographic Fourier domain diffuse correlation spectroscopy instrument

**DOI:** 10.1364/BOE.454346

**Published:** 2022-06-09

**Authors:** Edward James, Samuel Powell, Peter Munro

**Affiliations:** 1 Department of Medical Physics & Biomedical Engineering, University College London, London, WC1E 6BT, UK; 2 Faculty of Engineering, The University of Nottingham, University Park, Nottingham, NG7 2RD, UK

## Abstract

We have previously demonstrated a novel interferometric multispeckle Fourier domain diffuse correlation spectroscopy system that makes use of holographic camera-based detection, and which is capable of making *in vivo* pulsatile flow measurements. In this work, we report on a systematic characterisation of the signal-to-noise ratio performance of our system. This includes demonstration and elimination of laser mode hopping, and correction for the instrument’s modulation transfer function to ensure faithful reconstruction of measured intensity profiles. We also demonstrate a singular value decomposition approach to ensure that spatiotemporally correlated experimental noise sources do not limit optimal signal-to-noise ratio performance. Finally, we present a novel multispeckle denoising algorithm that allows our instrument to achieve a signal-to-noise ratio gain that is equal to the square root of the number of detected speckles, whilst detecting up to ∼1290 speckles in parallel. The signal-to-noise ratio gain of 36 that we report is a significant step toward mitigating the trade-off that exists between signal-to-noise ratio and imaging depth in diffuse correlation spectroscopy.

## 1. Introduction

Diffuse correlation spectroscopy (DCS) is a non-invasive optical imaging modality that can be used to measure cerebral blood flow (CBF) in real-time [1]. It has important potential applications in clinical monitoring [2], as well as in neuroscience and the development of a noninvasive brain-computer interface [3]. However, one of the limitations of DCS is that a trade-off exists between the signal-to-noise ratio (SNR) and imaging depth, and thus brain specificity, of this technique [4]. This is because an increase in imaging depth requires the use of larger source-detector separation (SDS) distances, which result in more photon losses due to absorption and scattering, and a subsequent decrease in SNR. An increase in imaging depth also results in the accumulation of more phase shifts due to dynamic scattering events, which results in a loss of coherence and SNR. Additionally, as DCS is a diffuse optical technique, it is limited by a lack of inherent depth discrimination within the illuminated region of each source-detector pair, and the CBF signal is therefore also prone to contamination by the extracerebral tissues which the light traverses [5].

The investigation of novel approaches to improve the sensitivity of DCS to CBF has therefore recently attracted interest from several research groups. Techniques including multispeckle detection strategies [3,6,7], time-domain DCS [8], DCS in the short-wave infrared region [9,10], interferometric approaches [4,11,12], and acousto-optic modulation [13] have all been proposed. Placing a particular emphasis on scalability, affordability, and robustness to ambient light, we have previously demonstrated a novel Fourier domain DCS (FD-DCS) instrument that makes use of heterodyne holographic camera-based detection, and which is capable of making *in vivo* pulsatile flow measurements [14,15]. The potential benefits of FD-DCS compared to conventional DCS are multiple: SNR that scales linearly with the square root of the number of camera pixels used, order of magnitude reduction in detector cost, robustness to the effects of ambient light, shot noise limited detection using off-axis holography [16], potential for detector scalability and sensor partitioning (which could facilitate tomographic and depth discrimination techniques [2,17]), and suitability to a range of design wavelengths (which could confer a further SNR advantage [9]).

Whilst our previous proof-of-concept work validated FD-DCS, we were unable to demonstrate the increase in SNR that the theory of multispeckle detection predicts. Therefore in this work, we report on a systematic characterisation of the SNR performance of our holographic FD-DCS system. We account for the effect of laser mode hopping on our coherent multiple camera frame technique, and also experimentally validate the inclusion of a model of our system’s modulation transfer function (MTF) into the measured data. By using spatiotemporal filtering as a validation tool, we can assess whether any given experimental setup produces limiting noise sources that compromise maximal SNR performance. The final contribution of this paper is a novel multispeckle denoising algorithm, the development of which has allowed us to remove spatiotemporally uncorrelated noise from the measured data, and which has also allowed us to demonstrate a linear relationship between SNR and the square root of the number of speckles detected. By bringing together the above four strategies, we achieve an SNR gain of 36 for our phantom experiments, for a flow parameter output rate of 8.2 Hz, when detecting over ∼ 1290 heterodyne speckles for our inexpensive camera-based detection system.

## 2. Theory and methods

**Fig. 1.**
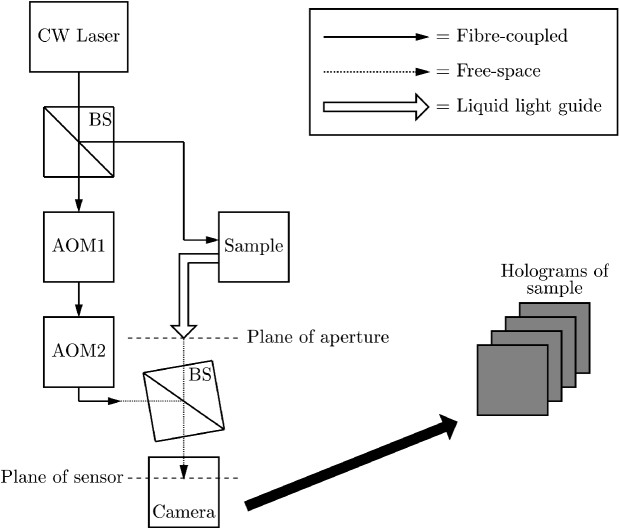
Schematic representation of the holographic FD-DCS system that is described in this paper. A continuous wave (CW) laser source is split into a reference arm and a sample arm in a fibre-coupled beamsplitter (BS). The reference arm is frequency shifted by a pair of acousto-optic modulators (AOM1 and AOM2). Light is collected from the sample in a reflectance mode geometry through the aperture of a liquid light guide. The two arms are recombined off-axis in a cube BS.

The theoretical framework and experimental setup of our holographic FD-DCS method are fully described in our previous publication [14]. Briefly, the technique employs a Mach-Zehnder interferometer where light from the sample arm interferes with frequency shifted light from the reference arm. A schematic representation of our experimental setup is shown in Fig. 1. Detecting the result of interference between the sample and the reference arms, for different reference light detuning frequencies, Δf , removes the need to detect very rapid intensity changes when frequency shifting is not used, as is required in conventional DCS experiments. This allows for a slower detector to be used, such as a relatively inexpensive camera. Thus, FD-DCS, which is inherently an interferometric technique, also lends itself well to multispeckle detection. Additionally, the interferometric measurement interrogates the electric field directly, rather than intensity, and therefore the Siegert relation, and the assumptions therein, do not constrain FD-DCS [18].

**Fig. 2.**
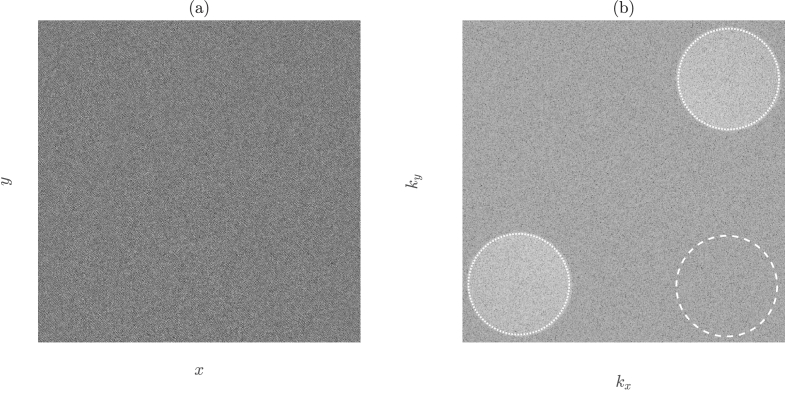
(a) Camera plane hologram, $H_{C}$ . (b) Reconstructed intensity hologram, $H_{R}$ . The two heterodyne gain terms, $S(\pm \Delta \omega ,k_{x},k_{y})$ , are masked by the dotted circles (which are a conjugate pair), the shot noise mask, $N(\Delta \omega ,k_{x},k_{y})$ , is depicted by the dashed circle.

According to the Wiener-Khinchin theorem, the first-order power spectral density (PSD) of the field fluctuations due to dynamic scatterers, $s_{1d}(\omega )$ , is the Fourier transform of the field autocorrelation function, $g_{1d}(\tau )$ [19–22],

(1) $$s_{1d}(\omega )=\int_{-\infty }^{+\infty }g_{1d}(\tau )\exp^{-i\omega \tau }\;d\tau ,$$

and thus an FD-DCS measurement and a conventional DCS measurement contain entirely equivalent information [23]. We sample the *unnormalised* first-order PSD, $S_{1}(\omega )$ , at a given reference arm detuning frequency, by first forming a camera plane hologram, $H_{C}$ , an example of which is shown in Fig. 2(a). For our lensless digital Fourier holography instrument [24,25], an intensity hologram, $H_{R}$ , is then reconstructed in the image plane by performing a 2D discrete Fourier transform (DFT) of $H_{C}$ [26,27]

(2) $$H_{R}=|F_{2D}(H_{C}){|}^{2},$$

an example of which is shown in Fig. 2(b). This reveals the twin holographic images of the heterodyne intensity of the speckle pattern that we wish to measure, which are a conjugate pair. Due to the off-axis recombination of the reference and sample arms in our instrument, the twin images are spatially separated in $H_{R}$ . A masking operation can then be implemented to take the sum over each of the two images and also to take the sum over a shot noise mask, which is located in one of the two ‘quiet’ corners of $H_{R}$ . The average pixel value in each mask is then obtained, which we denote by $\bar{S}(\pm \Delta \omega )$ for the two heterodyne masks, and $\bar{N}(\Delta \omega )$ for the shot noise mask. ${\bar{S}}_{1}(\pm \Delta \omega )$ may then be calculated for each heterodyne term as [19,28]

(3) $${\bar{S}}_{1}(\pm \Delta \omega )=\frac{\bar{S}(\pm \Delta \omega )}{\bar{N}(\Delta \omega )}-1.$$

Having made measurements of ${\bar{S}}_{1}(\pm \Delta \omega )$ at a range of detuning frequencies, we can then fit these measurements to an appropriate FD-DCS analytical model (taking into account both the type of motion and the modelled detection geometry) in order to extract a flow parameter measurement for the sample under consideration. DCS experiments typically report the effective Brownian diffusion coefficient, $D_{b}$ , as a flow parameter, which has been shown to be an effective surrogate for blood flow index (BFI) in a variety of tissue types *in vivo* [29]. For the phantom studies presented in this paper, the sample consists of a combined intralipid/deionised water optical phantom (Intralipid 20 %, Fresenius Kabi) with optical properties $\mu_{s}^{\prime }$ = 7.5 cm^-1^ and $\mu_{a}$ = 0.026 cm^-1^. A liquid light guide (LLG) with a 5.0 mm diameter core (Thorlabs, LLG5-4Z) is used to collect light from the sample in a reflection mode geometry, with the SDS distance set to 17.5 mm. Further details of our experimental setup can be found in Section 3 of [14]. The remainder of Section 2. describes the reconstruction and signal processing techniques that are used in this paper, the implementation of which is described in Section 3.

### 2.1 Modulation transfer function

Due to effect of the finite size of the camera pixels ( Δx,Δy ), the heterodyne detection efficiency within the space of $H_{R}$ ( $k_{x},k_{y}$ ) is given by the MTF of our lensless digital Fourier holography instrument [24,25,30]. Here ( $k_{x},k_{y}$ ) refers to spatial frequency, which is a function of the rate of sampling and the number of samples in the spatial domain [25]. For example, $k_{x}={\left(N\Delta x\right)}^{-1}$ , where N is the number of camera pixels in the x dimension. The MTF is the Fourier transform pair of the spatial distribution of a single pixel in the camera plane

(4) $$MTF(k_{x},k_{y})={\left|sinc(\sqrt{\alpha }\Delta xk_{x})sinc(\sqrt{\alpha }\Delta yk_{y})\right|}^{2},$$

where α is the camera pixel fill factor, and

(5) $$sinc(t)=\frac{sin(\pi t)}{\pi t}$$

is the normalised sinc function. We note that the each of the terms $\Delta xk_{x}$ and $\Delta yk_{y}$ in Eq. (4) is evaluated between ±0.5 across each of the two dimensions of the camera sensor [30]. An example of the MTF for α=0.72 is shown in Fig. 4(b). The MTF, which has rotational symmetry of order four, is centred on the reference beam (i.e., $k_{x}=k_{y}=0$ ) and results in increasing attenuation for increasing heterodyne spatial frequencies over the holographic twin images, but which does not affect the homodyne shot noise component [31]. We therefore update Eq. (3) to become

(6) $$S_{1}(\pm \Delta \omega ,k_{x},k_{y})=\frac{\frac{S(\pm \Delta \omega ,k_{x},k_{y})}{\bar{N}(\Delta \omega )}-1}{MTF(k_{x},k_{y})},$$

and we then proceed to take the average value within each heterodyne mask to make a measurement of ${\bar{S}}_{1}(\pm \Delta \omega )$ , which, as both of the heterodyne terms are identical for the holographic detection schemes described in this paper, we abbreviate to ${\bar{S}}_{1}(\Delta \omega )$ . We validate the inclusion of the MTF into the holographic reconstruction in Section 3.2. To the best of our knowledge, this is the first time that this inclusion has been validated in a digital holography experiment.

### 2.2 Singular value decomposition of holograms

The spatiotemporal filtering of holograms using a singular value decomposition (SVD) approach has recently been presented in the field of laser Doppler holography (LDH) in order to discriminate between the spatiotemporal characteristics of blood flow, and unwanted clutter such as bulk tissue motion, camera jitter, parasitic reflections, and other physical flaws in the recording channel [32,33]. The authors of the LDH technique achieved this by reconstructing holograms, having first performed an SVD of the holograms and setting the first $n_{c}$ singular values to zero.

Within the context of multispeckle interferometric DCS, a similar approach has also recently been presented by Robinson *et al.* [34]. These authors suggested that the largest singular values are also associated with movement artefacts and fluctuations in laser power, although the precise identity of the noise source is less important than the removal of a component of the measured data that is overly represented across the camera sensor, and which is therefore not due to the signal of an individual speckle.

The spatiotemporal filtering of holograms works by reshaping a series of $n_{t}$ consecutive holograms, of spatial dimensions $n_{x}\times n_{y}$ , into a 2D space-time matrix Q , which has dimensions $n_{x}n_{y}\times n_{t}$ . An SVD allows the matrix Q to be described as the sum of $n_{t}$ independent terms

(7) $$Q=\sum_{i=1}^{n_{t}}\lambda_{i}U_{i}V_{i}^{*},$$

where $\lambda_{i}$ are the singular values (ordered by decreasing value), $U_{i}$ are the left singular vectors (which correspond to space), $V_{i}$ are the right singular vectors (which correspond to time), and ^*^ denotes the complex conjugate transpose. The basis of the spatiotemporal filtering approach is that the highest magnitude singular values correspond to variations in Q with the strongest spatiotemporal correlations. Since speckle is expected to have weak spatiotemporal correlation, we can assume that strong spatiotemporal correlations in Q will be due to artefacts. In this work we propose to remove spatiotemporal clutter owing to channel noise in our experimental setup, that may be caused by laser instability and reflections at optical interfaces, for example. We do this by setting the first $n_{c}$ singular values to zero, and reconstructing Q using this updated vector of singular values. We use spatiotemporal filtering as a validation tool against which to benchmark the SNR performance of any given experimental setup, and we demonstrate this in Section 3.3.

### 2.3 Multispeckle detection noise in digital holography

Noise due to detector nonidealities will have an impact on the SNR performance of a multispeckle detection system [35], and in this paper we demonstrate a novel algorithm to effectively remove this noise from the measured data. In principle, we do this by first implementing a spatial sorting of the $S_{1}$ data within each reconstructed hologram, each of which is one in a series of independent and identically distributed random variables. This means that any temporal variation that exists between sorted holograms is due to both sampling noise, which is inherent to the speckle pattern that we wish to measure, and also detection noise. We can then apply a temporal filter to the sorted data to remove this noise. As detector noise occurs as white noise in each camera plane hologram, its DFT is effectively a random walk and can be assumed to have speckle-like statistics, and therefore it can be treated as an additional speckle-like noise in each reconstructed hologram [36]. Thus we propose that speckle reduction techniques can be adapted to remove detector noise from the sorted $S_{1}$ data. In this paper we propose median filtering, which has previously been employed to remove speckle noise from reconstructed holograms of static objects [37]. Having median filtered the sorted data, the initial sorting can then be reversed in order to restore the random nature of spatial speckle sampling. This algorithm is completely described in Section 3.4, where we also demonstrate and validate its inclusion into our signal processing pipeline.

## 3. Experiments and results

### 3.1 Mode hopping

**Fig. 3.**
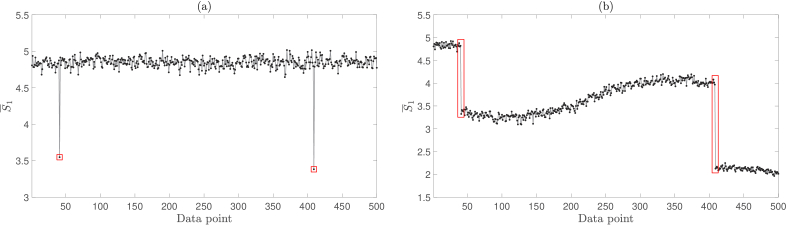
(a) Negative going outliers in ${\bar{S}}_{1}$ data (highlighted by the red squares). (b) Using an alternative temporal filtering strategy reveals discontinuities in intensity, which suggests that these outliers could be correlated with mode hopping (highlighted by the red rectangles).

Interferometric techniques inherently rely on splitting a light source into sample and reference arms. In our experimental setup, we use a 75:25 fibre-coupled beamsplitter (Thorlabs, 1x2 75:25 narrowband coupler, TN785R3A1) to form a sample arm and a reference arm, with an insertion loss of 1.28 dB and 6.09 dB, respectively (a further insertion loss of 1.09 dB is incurred on the reference arm due to the AOMs). However, we have found that back reflections from this beamsplitter into the laser cavity induce mode hopping that has deleterious effects on our temporal filtering strategy, as is confirmed later in this section. These effects are visible as negative going outliers in reconstructed ${\bar{S}}_{1}$ data [Fig. 3(a)], and occur at a rate of one in every 250 data points in this figure. Even though these outliers occur infrequently in this validation dataset, and could therefore easily be ignored, this would not be possible when detecting at the fast $D_{b}$ parameter output rates that are required to resolve pulsatile flow *in vivo*, which limit the number of ${\bar{S}}_{1}$ values used to fit per $D_{b}$ measurement.

The data presented in Fig. 3(a) were acquired using a DC subtraction temporal filtering method (analogous to the approach recently presented in [4]), in which the camera plane hologram, $H_{C}$ , is constructed as the difference of two successive images

(8) $$H_{C}=I_{n}-I_{n+1},$$

which serves as a high pass filter that removes the contribution of what we assume to be a temporally static contribution from the reference beam [16]. However, we hypothesise that if the laser were to mode hop between two successive images, then $H_{c}$ would be formed from two mutually incoherent images, which would result in an artefactual increase in $\bar{N}$ , with a subsequent decrease in ${\bar{S}}_{1}$ , according to Eq. (6). In order to test this theory, we used an alternative temporal filtering strategy

(9) $$H_{C}=I_{n}-I_{1},$$

where n≠1 , and we thus remove the contribution of the reference beam as it is recorded in the *first* camera frame of a measured series. The results of this analysis, shown in Fig. 3(b), reveal discontinuities in intensity, which suggests that these outliers could be correlated with mode hopping (this behaviour can also be demonstrated using an SVD approach - see Section 3.3).

The light source in our system is a single mode diode laser operating at 785 nm (Toptica, iBeam Smart 785-S-WS), which incorporates a ∼ 35 dB optical isolator fitted at the laser head to minimise back reflections into the laser cavity, and which has an insertion loss of 1.3 dB. Back reflections from optical interfaces can cause the laser to mode hop unpredictably [38], and even with the use of a single-stage optical isolator it is still possible to encounter back reflections into the laser. By employing the laser manufacturer’s proprietary feedback induced noise eraser (FINE) feature, we were able to eliminate the outlying data points demonstrated in Fig. 3(a), but at the expense of decreasing the measured ${\bar{S}}_{1}$ values and thus introducing noise into the $D_{b}$ measurement. By trimming the laser head to decrease back reflections into the main laser cavity, as well as incorporating a second optical isolator (Thorlabs, IO-F-780APC, 1.1 dB insertion loss) to achieve ∼ 71 dB of total optical isolation at the laser head, we are able to eliminate these outlying data points without employing FINE. Therefore, all three of FINE, laser head trimming, and dual-stage optical isolation were used as diagnostic tools to demonstrate the presence of mode hopping, but only the latter two were implemented as a solution in our experimental setup.

### 3.2 MTF correction

**Fig. 4.**
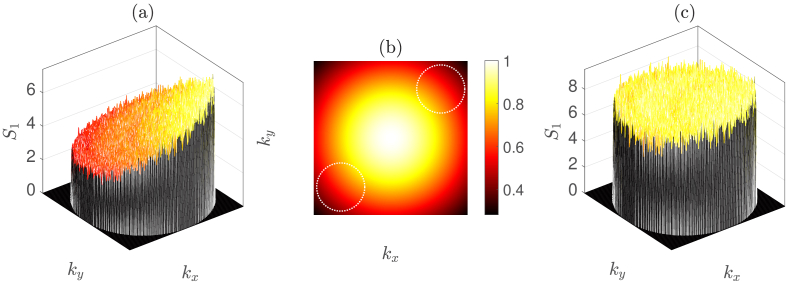
(a) Reconstructed average of 500 $S_{1}$ images without MTF correction. (b) MTF with α=0.72 , the white dotted circles indicate the location of the twin holographic images, which lie in a common plane. (c) Reconstructed average of 500 $S_{1}$ images with MTF correction.

**Fig. 5.**
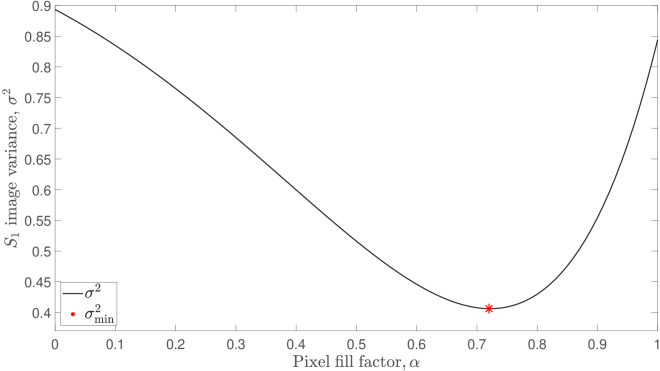
Choosing a value of α=0.72 minimises the variance, $\sigma_{min}^{2}$ , and thus maximises the flatness, of the average reconstructed $S_{1}$ image.

As our optical phantom is spatially invariant and has been imaged through the spatially incoherent core of an LLG of length 1.2 m, we expect to reconstruct a flat profile in Fig. 4(a), which shows the average intensity of 500 reconstructed $S_{1}$ images. However, the MTF of our instrument (see Section 2.1) causes a distortion artefact whereby higher spatial frequencies are more strongly attenuated. By minimising the variance, $\sigma^{2}$ , in the reconstructed $S_{1}$ image, for values of α in the range [0,1] , we can determine the α value for our experimental setup to be 0.72, as is shown in Fig. 5. The manufacturer of our camera (FLIR, BFS-U3-16S2M-CS) reports a camera pixel fill factor of 1.00, due to the microlenses that are used in the sensor array. The use of a microlens array will increase the light detection efficiency of the sensor; however, this does not take into account the optical aberrations of the microlenses that are relevant to our *imaging* application. Additionally, our camera has a maximum quantum efficiency over visible wavelengths, and its microlenses will therefore have a wavelength response that is not designed for the near infrared. By modelling a value of α=0.72 [Fig. 4(b)], we optimise the flatness of the average reconstructed $S_{1}$ image in Fig. 4(c), and thus correct for the distortion artefact caused by the MTF of the instrument. We note that this optimisation process can be customised to the features of any particular experimental setup, and that other appropriate optimisation targets for our experimental design could include radial symmetry, or the gradient of the radial average.

### 3.3 Spatiotemporal filtering and laser output power

**Fig. 6.**
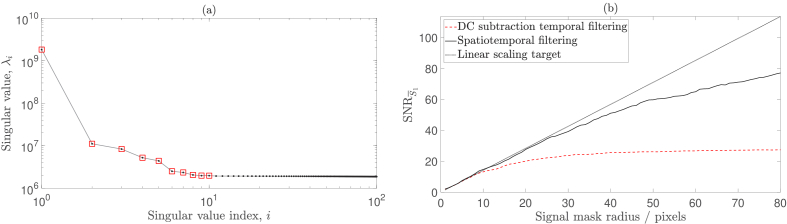
120 mW laser output power. (a) Singular values that result from the SVD of Q . The first 10 singular values (highlighted in red) are elevated and thus correlated with spatiotemporal noise. (b) Spatiotemporal filtering (plotted in solid black) results in an improvement in SNR performance compared to DC subtraction temporal filtering alone (plotted in dashed red).

We have previously demonstrated that the SNR of our ${\bar{S}}_{1}$ measurement does not scale linearly with the square root of the number of speckles detected when using a DC subtraction temporal filtering strategy [14,15], as is shown by the red dashed line in Fig. 6(b) for Δf = 0.1 Hz. Here we define the SNR in ${\bar{S}}_{1}$ to be the ratio of the mean value, μ , to the standard deviation, σ , of a sample of ${\bar{S}}_{1}$ values

(10) $${\text{SNR}}_{{\bar{S}}_{1}}=\frac{\mu ({\bar{S}}_{1})}{\sigma ({\bar{S}}_{1})},$$

over N repeats. For this experiment we use 501 camera plane holograms, which yields a value of N=500 for DC subtraction temporal filtering, and note that our laser is being driven at its maximum rated output power of 120 mW. By varying the size of the signal mask in the holographic reconstruction process, we can effectively control the number of speckles that contribute to each ${\bar{S}}_{1}$ measurement. In the absence of detector noise, and other experimental noise sources, the measurement SNR should be given by speckle statistics, i.e., the SNR of a speckle detection instrument should scale linearly with the square root of the number of detected speckles [35,39].

We propose to remove any spatiotemporally correlated experimental noise sources that may exist within this dataset using the SVD approach [32–34] that was introduced in Section 2.2. Q is formed from 501 camera plane holograms, each of spatial dimensions 512×512 pixels. Thus, the dimensions of Q are 262144×501 . We compute all 501 singular values of this matrix, the first 100 of which are shown in Fig. 6(a). The first 10 singular values are elevated due to spatiotemporally correlated noise, and we therefore use a threshold value of $n_{c}=10$ . Speckle has inherently weak spatiotemporal correlation, and we make use of this fundamental property by reconstructing Q having set the first $n_{c}$ singular values equal to zero. As the SVD step has already implemented temporal filtering, this allows us to form $H_{C}$ using single frame holography, i.e.,

(11) $$H_{C}=I_{n},$$

and we then proceed to reconstruct each $H_{R}$ according to Eq. (2). We then repeat the ${\text{SNR}}_{{\bar{S}}_{1}}$ analysis for this spatiotemporally filtered data and find that the SNR performance is closer to the linear scaling target, as is depicted in Fig. 6(b).

**Fig. 7.**
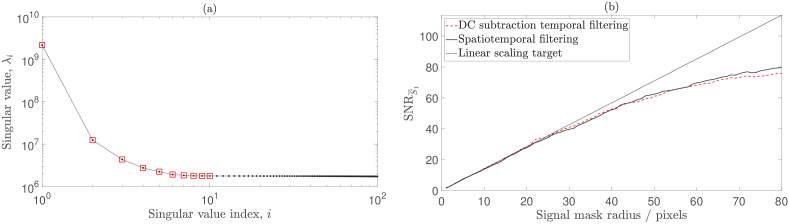
100 mW laser output power. (a) Singular values that result from the SVD of Q . The first 10 singular values (highlighted in red) are elevated and thus correlated with spatiotemporal noise. (b) Spatiotemporal filtering (plotted in solid black) results in a similar SNR performance to DC subtraction temporal filtering alone (plotted in dashed red).

With a view to characterising the source of the noise that has been removed by this SVD step, we repeated the above analysis on data acquired using a reduced laser output power of 100 mW, the results of which are shown in Fig. 7. This time, the spatiotemporal filtering approach results in similar SNR performance to the DC subtraction temporal filtering technique. We can therefore conclude that by reducing the laser output power, we have removed high frequency clutter from the measured data that is outside the stopband of a DC subtraction temporal filter. This can also be appreciated as a reduction in magnitude of some of the first 10 singular values in Fig. 7(a), compared to Fig. 6(a). Furthermore, as spatiotemporal filtering and DC subtraction temporal filtering offer similar SNR performance for this dataset, we can conclude that they have similar efficacy at removing low frequency clutter, which is within the stopband of a DC subtraction temporal filter.

As was also demonstrated by Puyo *et al.* [32], we have found that SVD provides a more robust basis than Fourier space to filter clutter from holograms. This is because high frequency clutter cannot be effectively removed using high pass temporal filtering alone. As this clutter is removed by decreasing the laser output power, it may be that the clutter is due to reflections that occur at optical interfaces within the experimental setup [38,40]. Indeed, inspection of the temporal singular vectors associated with elevated singular values reveals the presence of mode hopping (when dual-stage optical isolation is not used) and beat notes (when using a laser output power of 120 mW). It may also be that when pumping the laser at its maximum rated output power, phenomena such as increased spontaneous emission and technical noise (e.g., vibrations of the laser resonator, excess noise from the pump source, or temperature fluctuations) contribute noise to the measured data [41]. However, we use spatiotemporal filtering as a validation tool, rather than a final solution to implement in our signal processing pipeline, and therefore the precise identification of the sources of noise that spatiotemporal filtering removes is not imperative. The conclusion that DC subtraction temporal filtering, together with a sub-maximal laser output power, provides equivalent SNR performance to spatiotemporal filtering is key to validating our choice of DC subtraction as a temporal filtering strategy.

For these validation datasets, we have the luxury of computing singular values over a time-stack of $n_{t}=501$ camera frames. Using this approach when detecting *in vivo* pulsatile flow rates places significant restrictions on the hardware that is used. For example, Puyo *et al.* [32] used a value of $n_{t}=1024$ for their LDH technique, which was made possible by using a camera operating at a frame rate of 75 kHz. A DC subtraction temporal filtering strategy requires a minimum of only two camera frames, and is therefore a more appropriate choice for our experimental setup, which uses a camera operating at 200 Hz for *in vivo* experiments.

### 3.4 Novel multispeckle denoising algorithm

The remaining sources of noise demonstrably have no spatiotemporal correlation, and are therefore particularly challenging to remove, especially as the signal itself also has no spatiotemporal correlation. In Fig. 4(d) of [35], Xu *et al.* observed a similar phenomenon to that which we present in Fig. 7(b) of this paper. These authors postulated that the experimental SNR does not reach the predicted theoretical linear relationship with the square root of the number of detected speckles due to experimental imperfections, such as detector noise. The sources of noise that exist in a holographic reconstruction in lensless digital Fourier holography have been discussed in [27,36], and these include detector nonidealities (such as quantisation noise, read noise, and pixel nonuniformity noise) and noise due to superimposed diffraction patterns caused by dust particles in the interferometric path.

**Fig. 8.**
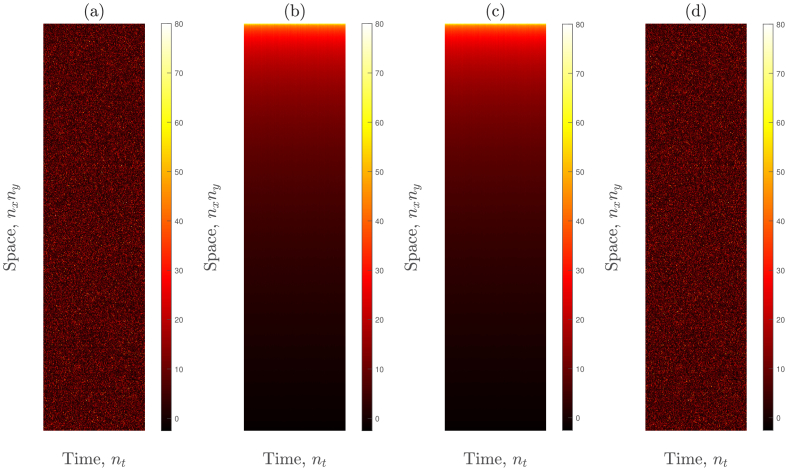
Novel multispeckle denoising algorithm. (a) The 2D space-time matrix, R . (b) Each column of R is sorted into ascending order. (c) R is then median filtered using a [1 × 3] neighbourhood. (d) The sorting is reversed. N.B. The maximum $S_{1}$ value in matrices (a) and (b) is 163, and the maximum $S_{1}$ value in matrices (c) and (d) is 116; however, we have used a high threshold of 80 in each subplot of this figure to aid visualisation.

Here we present a method that allows us to remove this noise from the measured data. We start by constructing a 2D space-time matrix, as described in Sections 2.2 and 3.3, but this time we reshape *reconstructed* holograms (which have undergone DC subtraction temporal filtering), and we denote this matrix R . For this example, we use the same dataset that has been analysed in Fig. 7 (i.e., a laser output power of 100 mW and a detuning frequency of 0.1 Hz). There are 20081 camera pixels within each of the signal masks shown in Fig. 2(b), and we reshape the values within one of these signal masks into a column vector. This is then repeated for $n_{t}=500$ reconstructed holograms, and the resulting 500 column vectors are horizontally concatenated to form R , which has dimensions 20081×500 , an example of which is shown in Fig. 8(a). The formation of R does not alter the $S_{1}$ values within each reconstructed hologram, and, since this matrix is yet to be denoised, we refer to it as control data.

In theory, each column of R represents the same distribution of $S_{1}$ values, but which has been independently randomised due to the nature of spatial speckle sampling, and which has also been contaminated with both sampling noise and measurement noise. The next step of the multispeckle denoising algorithm involves independently sorting the elements of each column of R into ascending order, as is shown in Fig. 8(b). Having removed the inherently random nature of the *spatial* sampling of speckle within each column, we can now proceed to *temporal* filtering between columns to remove noise. We do this by filtering the sorted matrix using a [1 × n ] neighbourhood (which refers to the space and time axes of R , respectively) and we choose a median filter, as was discussed in Section 2.3, with a value of n=3 . We are motivated to use a low value of n so as not to compromise the temporal resolution of the measurement, and we have found that n=3 is the lowest value of n that achieves the linear SNR scaling that multispeckle detection predicts [Fig. 10]. The results of median filtering the sorted matrix are shown in Fig. 8(c). We then reverse the sorting of each column, as is shown in Fig. 8(d), and we refer to this matrix as denoised data.

**Fig. 9.**
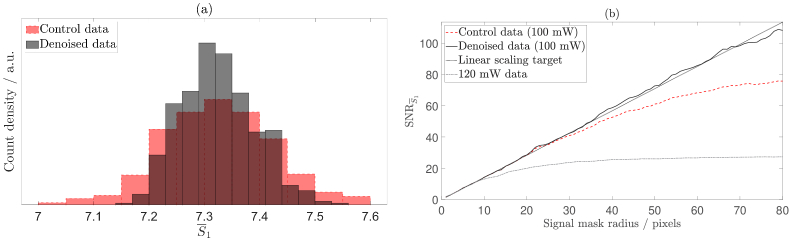
(a) Red and black histograms show the distribution of 500 ${\bar{S}}_{1}$ values for control and denoised data ( n=3 ), respectively. (b) Denoising achieves the theoretical linear scaling target for SNR performance, as shown by the black solid line. For effective comparison, SNR performance achieved using 120 mW laser output power and DC subtraction temporal filtering is shown by the grey dash-dotted line.

The distribution of the ${\bar{S}}_{1}$ values of each column of the control data are shown by the red histogram in Fig. 9(a), which also shows the distribution of the ${\bar{S}}_{1}$ values of each column of the denoised data by the black histogram. By applying our novel multispeckle denoising algorithm, we have reduced the variance of the data without disturbing its central tendency. 99.95 % of the noise that has been removed from this dataset has an absolute value less than the camera read noise (2.45 photoelectrons), and 99.99 % of the noise that has been removed has an absolute value less than the camera quantisation interval (5.73 photoelectrons).

**Fig. 10.**
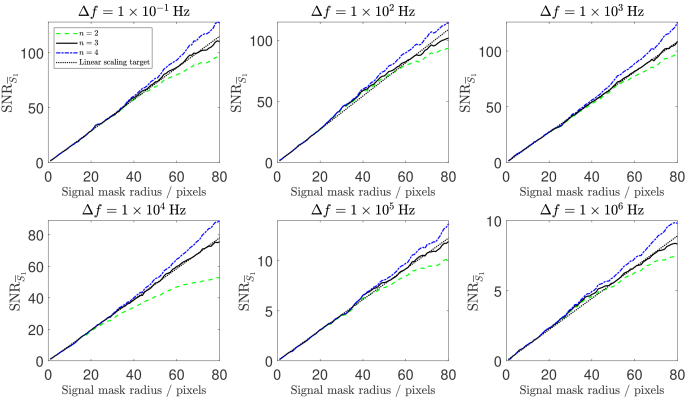
Denoising with n=3 achieves the theoretical linear scaling target for SNR performance at all six detuning frequencies for this dataset, as shown by the black solid line in each subplot. Denoising with n=4 outperforms the linear scaling target at a cost of decreased temporal resolution.

**Fig. 11.**
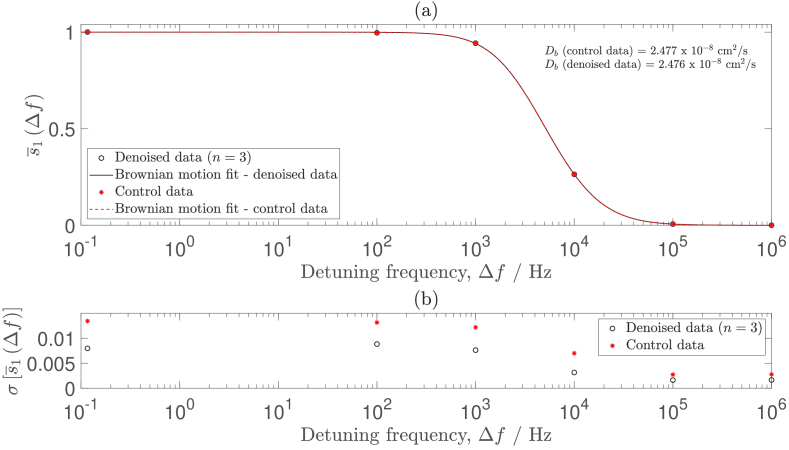
(a) Denoising does not corrupt the signal contained within the PSD measurement. The $D_{b}$ values fitted to control and denoised data are within 0.02 %, 0.02 %, and 0.01 % of each other for a Brownian motion fit, for values of n=2,3, and 4, respectively. (b) The standard deviation of the PSD measurement is decreased by denoising for all detuning frequencies.

We then reorder each of the columns of the denoised data back into the form of the native signal mask, and repeat the ${\text{SNR}}_{{\bar{S}}_{1}}$ analysis that is described in Section 3.3. The results of this are shown by the black solid line in Fig. 9(b), which demonstrates that the theoretical linear scaling target for SNR performance has been achieved. We repeat this validation for all six detuning frequencies for this dataset, as is shown in Fig. 10. Additionally, in order to verify that the denoising process does not corrupt the PSD measurement, we fit $D_{b}$ to both control and denoised data in Fig. 11, and confirm that the signal is unchanged by the denoising process.

**Fig. 12.**
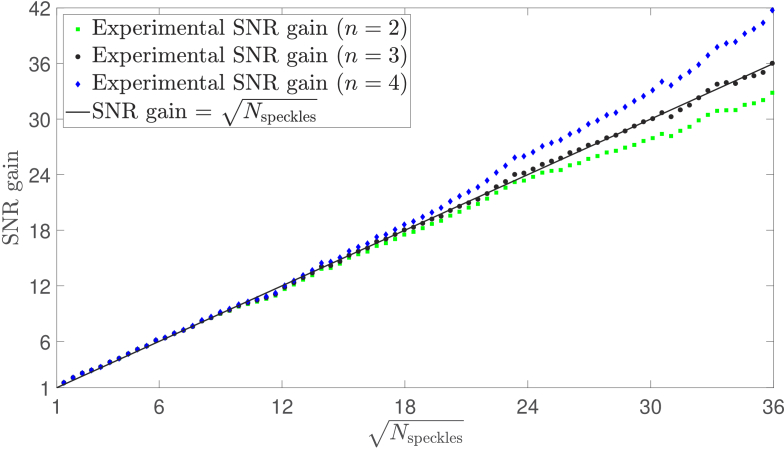
Experimental SNR gain for a detuning frequency of 1 kHz. Using a value of n=3 , the experimental data fit the theoretical prediction that SNR gain is equal to the square root of the number of detected speckles, $\sqrt{N_{speckles}}$ . Using a value of n=4 outperforms this linear prediction by reducing the independence of consecutive holograms.

Finally, we define the SNR gain of our multispeckle detection system to be the ratio of ${\text{SNR}}_{{\bar{S}}_{1}}$ achieved with multispeckle DCS to ${\text{SNR}}_{{\bar{S}}_{1}}$ achieved with single speckle DCS, using detectors with the same performance [3] and at the same detuning frequency. The geometry of our experimental setup has been described in our previous work [14], and for the observation distance used in the current dataset ( z=76.84 mm), a single speckle occupies 15.6 pixels on the camera sensor (which has a pixel size of 3.45 μ m), according to the relationship [42]

(12) $$S=\frac{{\left(\lambda z\right)}^{2}}{A_{\text{aperture}}},$$

where S is the speckle area, λ is the operating wavelength, and $A_{\text{aperture}}$ is the area of the aperture of the LLG. Figure 12 shows that, at a detuning frequency of 1 kHz and for a value of n=3 , the experimental SNR gain fits the theoretical prediction that SNR gain is equal to the square root of the number of detected speckles, and we find that this relationship is validated at all six measured detuning frequencies for this dataset. We achieve an SNR gain of 36 when detecting ∼ 1290 speckles in parallel.

## 4. Outlook and discussion

The current state-of-the-art in SNR performance achieved by a multispeckle DCS system is described by Sie *et al.* [3], who reported an SNR gain of 32 in a phantom study when detecting *homodyne* speckles using a 1024 pixel single-photon avalanche diode camera, with an SDS distance of 11.0 mm. We have achieved an SNR gain of 36 in a phantom study when detecting over ∼ 1290 *heterodyne* speckles in parallel, with an SDS distance of 17.5 mm, using a detector that is two orders of magnitude less expensive. Additionally, compared to homodyne DCS, heterodyne DCS has been shown to offer an SNR gain of ∼ 2 for phantom experiments [12].

An *in vivo* SNR gain of 16 has recently been reported in a DCS system that has a design wavelength of 1064 nm, and which uses superconducting nanowire single-photon detectors, with an SDS distance of 25.0 mm on the forehead of human subjects [10]. Although we have not reported *in vivo* results in this paper, this, together with optimisation of an *in vivo* probe, will form part of our future work. The *in vivo* data that we have previously presented [14] involved the capture of three camera frames at each detuning frequency (using a sub-maximal laser output power of 39 mW), with a subsequent $D_{b}$ frame rate of 10.8 Hz for our current experimental setup. We note that our multispeckle denoising algorithm requires the capture of four camera frames at each detuning frequency for n=3 , and doing so slows down the resulting $D_{b}$ frame rate to 8.2 Hz. However, this frame rate is still sufficiently fast to recover pulsatile information, and validating our denoising algorithm on *in vivo* data is therefore future work.

The value of n that is used in the denoising algorithm represents a trade-off between temporal resolution and denoising performance. Indeed, we note that by using a value of n>3 we can reduce the independence of consecutive holograms further, without perturbing the measured signal, thereby overcoming the linear SNR scaling limit imposed by sampling noise [Fig. 12], but at the cost of a decreased temporal resolution. For any given value of n , temporal averaging *without* sorting achieves the same magnitude SNR gain as temporal averaging *with* sorting, when evaluated at the maximum sampled mask radius. However, without sorting, the SNR gain does not scale as it should with the square root number of speckles, and therefore leaves noise sources unaccounted for. In this paper, we have found that median filtering with sorting, for a value of n=3 , yields the SNR statistics that we expect, and we have shown that this can be achieved by accounting for both spatiotemporally correlated noise sources and detector noise, which occurs as white noise in the camera plane.

Previous authors have noted that multispeckle detection introduces extreme sensitivity to motion artefacts of the multimode detector fibre [12], and it is therefore surprising that we have not identified noise due to movement artefact of the LLG in this SNR optimisation study. In addition to the experimental findings described in Section 3, preliminary investigations have shown that using a free-space propagation setup (i.e., bypassing the LLG) does not improve SNR performance. Gross described that the spatial filtering step of off-axis holography can be used to remove technical noise in the reference arm due to vibrations [43], and it is therefore possible that motion artefacts of the LLG in our experimental setup are removed in this manner, but for this to be the case the noise would need to be composed of predominantly low spatial frequencies. Further characterisation of the effects of motion on the transfer matrix of the LLG is therefore required in order to understand this further.

Although in our previous work we have shown that holographic FD-DCS yields an SNR advantage over conventional DCS when using an optical phantom with $\mu_{a}$ = 0.1 cm^-1^ [14], in the present study we have used a relatively low-absorption phantom with $\mu_{a}$ = 0.026 cm^-1^. This is the phantom that was used in our previous publication to demonstrate absolute equivalence between conventional DCS and holographic FD-DCS, as it allows for a greater range of experimental parameters, and we use it again here to characterise SNR performance. Absolute SNR will decrease with increasing sample absorption; however, we do not expect any change in the relationship between SNR gain and the square root of the number of detected speckles (which is the focus of this paper) when increasing sample absorption. The extrapolation of the findings of this paper to higher absorption samples therefore forms part of our future work. Further to this, investigating the effects of varying photon count rates and reference arm power levels on absolute SNR would be a useful further study.

The autocorrelation of faster blood flow will decorrelate more quickly, and therefore, for a given acquisition rate, conventional DCS will have an upper limit on the speed of blood flow that can be resolved. However, when operating in the Fourier domain, faster blood flows will have broader power spectra, which does not present a challenge to detection for our instrument. This suggests that FD-DCS may have an advantage over conventional DCS with regard to detecting faster flows, and further investigation into this hypothesis is warranted. A further potential advantage of FD-DCS is the ability to select which detuning frequencies to sample at, which may be beneficial when detecting deeper flow using larger SDS distances (DCS measurements of CBF require an SDS distance of ≥ 25 mm [12]). In conventional DCS, shorter time lags are more representative of photons that have travelled deeper into the sample [12], and techniques such as fitting early time lags and estimating the zero-lag derivative can enhance depth sensitivity [4]. Although we have not yet investigated SDS distances greater than 17.5 mm, doing so is part of our future work, in which we will also explore the preferential fitting of larger detuning frequencies (which can be specified arbitrarily using our instrument).

The computational processing requirements of holographic FD-DCS are high, especially when operating in real-time at fast $D_{b}$ frame rates. With a view to reducing the computational demand of conventional DCS experiments, deep learning techniques have recently been employed [44], resulting in a 23-fold increase in the speed of blood flow quantification. The application of deep learning techniques to holographic FD-DCS would be an interesting further study. We note that the generation of training data could be performed using the algorithms that we have presented for the generation of wide-field two-dimensional time-integrated dynamic speckle patterns [45], which would serve as a forward model for that which is detected on the sample arm of the instrument.

Finally, the SNR gain reported in this paper has the potential to facilitate the measurement of acousto-optically modulated DCS signals *in vivo*, which are weak at biologically safe power levels [46]. By operating in the Fourier domain, we obviate the need for high frame rate detection, thus making our low frame rate detection strategy suitable for this purpose. Therefore, our future work will also involve the development of an acousto-optically modulated FD-DCS analytical model, as well as an exploration of depth-resolved flow measurement strategies using this technique. An alternative approach to achieving depth discrimination, which facilitates the removal of extracerebral contamination, would be by extension to a superficial regression technique [2] or tomographic approach [17]. With further experimental effort it would be possible to measure multiple source-detector pairs on the same sensor (by using a spatially coherent fibre bundle or multiple detector fibres, for example), and these investigations also form part of our future work.

## 5. Summary and conclusions

The use of DC subtraction temporal filtering has been well described in the digital holography literature: it is a strategy that can achieve shot noise limited detection with only two camera frames. However, in Section 3.1 we documented the vulnerability of this technique to laser mode hopping, which, to the best of our knowledge, has not been reported in the literature before. Whilst the outliers caused by this vulnerability could easily be ignored when analysing validation datasets, this is not possible when detecting at the high parameter output rates that are necessary for *in vivo* detection, and thus it is preferable to eliminate them at source using hardware based techniques.

Whilst a model for the MTF of a lensless digital Fourier holography instrument is accepted within the relevant literature, its experimental validation has not, to the best of our knowledge, been reported before. In Sections 2.1 and 3.2, we therefore revised the reconstruction of an unnormalised PSD measurement using digital holography in order to include the MTF of the instrument. Although the MTF will not vary from camera frame to camera frame, and therefore does not affect absolute validation experiments, it does increase the variance of the data and therefore introduce noise. It is therefore important to correct for the MTF when optimising SNR performance.

In Section 2.2 we describe the removal of spatiotemporally correlated noise sources from holograms using SVD filtering, and we then implement this approach in Section 3.3. As well as being a useful tool to eliminate and characterise noise sources, we use this approach as a validation tool to ensure that source noise does not decrease SNR performance. Specifically, we find that using a sub-maximal laser source power is necessary to ensure the removal of source noise.

Having used SVD filtering to remove spatiotemporally correlated noise sources, we then introduced a novel multispeckle denoising algorithm to remove spatiotemporally uncorrelated noise sources in Section 2.3. This algorithm is implemented in Section 3.4, where it has yielded the demonstration of a linear relationship, and beyond, between SNR and the square root of the number of speckles detected, by allowing for the removal of both detector noise and sampling noise.

In conclusion, we have presented a systematic characterisation of the SNR performance of our holographic FD-DCS instrument. By bringing together the four methods detailed in this paper, we have achieved an SNR gain that it is equal to the square root of the number of measured speckles, for a flow parameter output rate of 8.2 Hz, using scalable low-cost camera-based detection. This represents a significant step toward improving the SNR of DCS measurements of blood flow, as well as improving the affordability of such a system.

## Data Availability

Data underlying the results presented in this paper are not publicly available at this time but may be obtained from the authors upon reasonable request.

## References

[1] Wang D. Parthasarathy A. B. Baker W. B. Gannon K. Kavuri V. Ko T. Schenkel S. Li Z. Li Z. Mullen M. T. Detre J. A. Yodh A. G., “Fast blood flow monitoring in deep tissues with real-time software correlators,” Biomed. Opt. Express 7(3), 776–797 (2016). 10.1364/BOE.7.000776 27231588 PMC4866455

[2] Selb J. Wu K.-C. Sutin J. Lin P.-Y. I. Farzam P. Bechek S. Shenoy A. Patel A. B. Boas D. A. Franceschini M. A. Rosenthal E. S., “Prolonged monitoring of cerebral blood flow and autoregulation with diffuse correlation spectroscopy in neurocritical care patients,” Neurophotonics 5(04), 1 (2018). 10.1117/1.NPh.5.4.045005 PMC6233866 30450363

[3] Sie E. J. Chen H. Saung E.-F. Catoen R. Tiecke T. Chevillet M. A. Marsili F., “High-sensitivity multispeckle diffuse correlation spectroscopy,” Neurophotonics 7(03), 035010 (2020). 10.1117/1.NPh.7.1.035010 32995362 PMC7519351

[4] Zhou W. Zhao M. Kholiqov O. Srinivasan V. J., “Multi-exposure interferometric diffusing wave spectroscopy,” Opt. Lett. 46(18), 4498–4501 (2021). 10.1364/OL.427746 34525031 PMC9612632

[5] Zhou W. Kholiqov O. Zhu J. Zhao M. Zimmermann L. L. Martin R. M. Lyeth B. G. Srinivasan V. J., “Functional interferometric diffusing wave spectroscopy of the human brain,” Sci. Adv. 7(20), eabe0150 (2021). 10.1126/sciadv.abe0150 33980479 PMC8115931

[6] Murali K. Varma H. M., “Multi-speckle diffuse correlation spectroscopy to measure cerebral blood flow,” Biomed. Opt. Express 11(11), 6699–6709 (2020). 10.1364/BOE.401702 33282518 PMC7687951

[7] Liu W. Qian R. Xu S. Chandra Konda P. Jönsson J. Harfouche M. Borycki D. Cooke C. Berrocal E. Dai Q. Wang H. Horstmeyer R., “Fast and sensitive diffuse correlation spectroscopy with highly parallelized single photon detection,” APL Photonics 6(2), 026106 (2021). 10.1063/5.0031225

[8] Sutin J. Zimmerman B. Tyulmankov D. Tamborini D. Wu K. C. Selb J. Gulinatti A. Rech I. Tosi A. Boas D. A. Franceschini M. A., “Time-domain diffuse correlation spectroscopy,” Optica 3(9), 1006–1013 (2016). 10.1364/OPTICA.1.001006 28008417 PMC5166986

[9] Carp S. A. Tamborini D. Mazumder D. Wu K.-C. Robinson M. B. Stephens K. A. Shatrovoy O. Lue N. Ozana N. Blackwell M. H. Franceschini M. A., “Diffuse correlation spectroscopy measurements of blood flow using 1064 nm light,” J. Biomed. Opt. 25(09), 097003 (2020). 10.1117/1.JBO.25.9.097003 PMC7522668 32996299

[10] Ozana N. Zavriyev A. I. Mazumder D. Robinson M. B. Kaya K. Blackwell M. H. Carp S. A. Franceschini M. A., “Superconducting nanowire single-photon sensing of cerebral blood flow,” Neurophotonics 8(03), 035006 (2021). 10.1117/1.NPh.8.1.035006 34423069 PMC8373637

[11] Zhou W. Kholiqov O. Chong S. P. Srinivasan V. J., “Highly parallel, interferometric diffusing wave spectroscopy for monitoring cerebral blood flow dynamics,” Optica 5(5), 518–527 (2018). 10.1364/OPTICA.5.000518 30417035 PMC6226099

[12] Robinson M. B. Boas D. A. Sakadžic S. Franceschini M. A. Carp S. A., “Interferometric diffuse correlation spectroscopy improves measurements at long source-detector separation and low photon count rate,” J. Biomed. Opt. 25(09), 097004 (2020). 10.1117/1.JBO.25.9.097004 PMC7525153 33000571

[13] Robinson M. B. Carp S. A. Peruch A. Boas D. A. Franceschini M. A. Sakadžić S., “Characterization of continuous wave ultrasound for acousto-optic modulated diffuse correlation spectroscopy (AOM-DCS),” Biomed. Opt. Express 11(6), 3071–3090 (2020). 10.1364/BOE.390322 32637242 PMC7316011

[14] James E. Powell S., “Fourier domain diffuse correlation spectroscopy with heterodyne holographic detection,” Biomed. Opt. Express 11(11), 6755–6779 (2020). 10.1364/BOE.400525 33282522 PMC7687971

[15] James E Powell S, “Diffuse correlation spectroscopy in the Fourier domain with holographic camera-based detection, in *Dynamics and Fluctuations in Biomedical Photonics XVII* , vol. 11239, Tuchin V. V. Leahy M. J. Wang R. K., eds., International Society for Optics and Photonics (SPIE, 2020), pp. 29–35.

[16] Gross M. Atlan M. Absil E., “Noise and aliases in off-axis and phase-shifting holography,” Appl. Opt. 47(11), 1757–1766 (2008). 10.1364/AO.47.001757 18404173

[17] Zhou C. Yu G. Furuya D. Greenberg J. H. Yodh A. G. Durduran T., “Diffuse optical correlation tomography of cerebral blood flow during cortical spreading depression in rat brain,” Opt. Express 14(3), 1125–1144 (2006). 10.1364/OE.14.001125 19503435

[18] Xu J. Jahromi A. K. Brake J. Robinson J. E. Yang C., “Interferometric speckle visibility spectroscopy (ISVS) for human cerebral blood flow monitoring,” APL Photonics 5(12), 126102 (2020). 10.1063/5.0021988

[19] Magnain C. Castel A. Boucneau T. Simonutti M. Ferezou I. Rancillac A. Vitalis T. Sahel J. A. Paques M. Atlan M., “Holographic laser doppler imaging of microvascular blood flow,” J. Opt. Soc. Am. A 31(12), 2723–2735 (2014). 10.1364/JOSAA.31.002723 25606762

[20] Brown J. C., “Optical correlations and spectra,” Am. J. Phys. 51(11), 1008–1011 (1983). 10.1119/1.13359

[21] Atlan M. Desbiolles P. Gross M. Coppey-Moisan M., “Parallel heterodyne detection of dynamic light-scattering spectra from gold nanoparticles diffusing in viscous fluids,” Opt. Lett. 35(5), 787–789 (2010). 10.1364/OL.35.000787 20195353

[22] Goodman J., *Statistical Optics* (Wiley, 2015), 2nd ed.

[23] Boas D. A. Yodh A. G., “Spatially varying dynamical properties of turbid media probed with diffusing temporal light correlation,” J. Opt. Soc. Am. A 14(1), 192–215 (1997). 10.1364/JOSAA.14.000192

[24] Wagner C. Seebacher S. Osten W. Jüptner W., “Digital recording and numerical reconstruction of lensless fourier holograms in optical metrology,” Appl. Opt. 38(22), 4812–4820 (1999). 10.1364/AO.38.004812 18323970

[25] Kreis T. M., “Frequency analysis of digital holography,” Opt. Eng. 41(4), 771–778 (2002). 10.1117/1.1458551

[26] Schnars U. Jüptner W. P. O., “Digital recording and numerical reconstruction of holograms,” Meas. Sci. Technol. 13(9), R85–R101 (2002). 10.1088/0957-0233/13/9/201

[27] Schnars U. Falldorf C. Watson J. Jüptner W., *Digital Holography and Wavefront Sensing - Principles, Techniques and Applications* (Springer, 2010), 2nd ed.

[28] Gross M. Goy P. Forget B. C. Atlan M. Ramaz F. Boccara A. C. Dunn A. K., “Heterodyne detection of multiply scattered monochromatic light with a multipixel detector,” Opt. Lett. 30(11), 1357–1359 (2005). 10.1364/OL.30.001357 15981532

[29] Durduran T. Choe R. Baker W. B. Yodh A. G., “Diffuse optics for tissue monitoring and tomography,” Rep. Prog. Phys. 73(7), 076701 (2010). 10.1088/0034-4885/73/7/076701 26120204 PMC4482362

[30] Verpillat F. Joud F. Atlan M. Gross M., “Digital holography at shot noise level,” J. Display Technol. 6(10), 455–464 (2010). 10.1109/JDT.2010.2044366

[31] Goodman J. W., *Introduction to Fourier Optics* (W.H. Freeman, 2017), 4th ed.

[32] Puyo L. Paques M. Atlan M., “Spatio-temporal filtering in laser Doppler holography for retinal blood flow imaging,” Biomed. Opt. Express 11(6), 3274–3287 (2020). 10.1364/BOE.392699 32637254 PMC7316027

[33] Atlan M Touminet A Andal T Puyo L Pâques M, “Image-based digital motion and aberration compensation in laser Doppler holography of the eye fundus (Conference Presentation), in *Adaptive Optics and Wavefront Control for Biological Systems VI* , vol. 11248, Bifano T. G. Gigan S. Ji N., eds., International Society for Optics and Photonics (SPIE, 2020).

[34] Robinson M. B. Carp S Peruch A Ozana N Franceschini M, “High framerate, InGaAs camera for interferometric diffuse correlation spectroscopy (iDCS) beyond the water peak (Conference Presentation), in *Dynamics and Fluctuations in Biomedical Photonics XVIII* , vol. 11641, Tuchin V. V. Leahy M. J. Wang R. K., eds., International Society for Optics and Photonics (SPIE, 2021).

[35] Xu J. Jahromi A. K. Yang C., “Diffusing wave spectroscopy: A unified treatment on temporal sampling and speckle ensemble methods,” APL Photonics 6(1), 016105 (2021). 10.1063/5.0034576

[36] Pandey N. Hennelly B., “Quantization noise and its reduction in lensless Fourier digital holography,” Appl. Opt. 50(7), B58–B70 (2011). 10.1364/AO.50.000B58 21364713

[37] Garcia-Sucerquia J. Ramírez J. A. H. Prieto D. V., “Reduction of speckle noise in digital holography by using digital image processing,” Optik 116(1), 44–48 (2005). 10.1016/j.ijleo.2004.12.004

[38] Biswas A. Moka S. Muller A. Parthasarathy A. B., “Fast diffuse correlation spectroscopy with a low-cost, fiber-less embedded diode laser,” Biomed. Opt. Express 12(11), 6686–6700 (2021). 10.1364/BOE.435136 34858674 PMC8606156

[39] Varma H. M. Valdes C. P. Kristoffersen A. K. Culver J. P. Durduran T., “Speckle contrast optical tomography: A new method for deep tissue three-dimensional tomography of blood flow,” Biomed. Opt. Express 5(4), 1275–1289 (2014). 10.1364/BOE.5.001275 24761306 PMC3986001

[40] Colomb T. Dahlgren P. Beghuin D. Cuche E. Marquet P. Depeursinge C., “Polarization imaging by use of digital holography,” Appl. Opt. 41(1), 27–37 (2002). 10.1364/AO.41.000027 11900443

[41] Peng J. Yu H. Liu J. Cao Y. Zhang Z. Sun L., “Principles, measurements and suppressions of semiconductor laser noise - a review,” IEEE J. Quantum Electron. 57(5), 1–15 (2021). 10.1109/JQE.2021.3093885

[42] Goodman J., *Speckle Phenomena in Optics - Theory and Applications* (SPIE, 2020), 2nd ed.

[43] Gross M., “Heterodyne holography with full control of both the signal and reference arms,” Appl. Opt. 55(3), A8–A16 (2016). 10.1364/AO.55.0000A8 26835961

[44] Poon C.-S. Long F. Sunar U., “Deep learning model for ultrafast quantification of blood flow in diffuse correlation spectroscopy,” Biomed. Opt. Express 11(10), 5557–5564 (2020). 10.1364/BOE.402508 33149970 PMC7587273

[45] James E. Powell S. Munro P., “Simulation of statistically accurate time-integrated dynamic speckle patterns in biomedical optics,” Opt. Lett. 46(17), 4390–4393 (2021). 10.1364/OL.435812 34470023

[46] Gunther J. Andersson-Engels S., “Review of current methods of acousto-optical tomography for biomedical applications,” Front. Optoelectron. 10(3), 211–238 (2017). 10.1007/s12200-017-0718-4

